# Research Ethics Boards: Size, Not Money

**DOI:** 10.1371/journal.pmed.0030457

**Published:** 2006-10-31

**Authors:** Joal Hill

I read with interest the debate about for-profit versus non-profit institutional review boards (IRBs) [1], but was disappointed that no one addressed the ability (or inability) of for-profit IRBs to review studies with the local context of research subjects in mind and then monitor what actually occurs during the consent process throughout the research trial.

To my mind the “bigness” of for-profit IRBs may be more of an impediment in protecting research subjects than their inherent conflict of interest. Our IRB has reviewed consent forms approved by central/for-profit IRBs that contained obvious errors such as schemas that did not match protocol narrative and use of eight point font in a study of geriatric subjects. Even when the initial review is outstanding, it seems a practical impossibility for a single IRB to provide meaningful monitoring of the actual consent process and implementation of the protocol at sites throughout the country. The greater “efficiency” of for-profit IRBs is only a meaningful benefit if increased speed can be shown not to occur at the expense of careful review of consent forms, real understanding of the local research context, and a commitment to audit the informed consent process throughout the study for the protection of research subjects, including ongoing education and advice for researchers and their teams.

This is not to say that all local IRBs perform this function as they should, but it does seem almost impossible for one IRB to perform local review and oversight for research sites around the nation in a way that really makes a difference for the men, women, and children who give of their time and their bodies so that society can benefit.
